# A greedy assist-as-needed controller for end-effect upper limb rehabilitation robot based on 3-DOF potential field constraints

**DOI:** 10.3389/frobt.2024.1404814

**Published:** 2024-10-14

**Authors:** Yue Lu, Zixuan Lin, Yahui Li, Jinwang Lv, Jiaji Zhang, Cong Xiao, Ye Liang, Xujiao Chen, Tao Song, Guohong Chai, Guokun Zuo

**Affiliations:** ^1^ Cixi Biomedical Research Institute, Wenzhou Medical University, Ningbo, China; ^2^ Ningbo Institute of Materials Technology and Engineering, Chinese Academy of Sciences, Ningbo, China; ^3^ Ningbo Cixi Institute of Biomedical Engineering, Ningbo, China; ^4^ University of Chinese Academy of Sciences, Beijing, China; ^5^ Department of Oral and Maxillofacial Surgery, Center of Stomatology, Xiangya Hospital, Central South University, Changsha, China; ^6^ Department of Geriatrics, The First Affliated Hospital of Zhejiang Chinese Medical University (Zhejiang Provincial Hospital of Chinese Medicine), Hangzhou, China

**Keywords:** Assist-as-needed (AAN), 3-DOF potential field, radial basis function (RBF) network, human-robot interaction, rehabilitation robot

## Abstract

It has been proven that robot-assisted rehabilitation training can effectively promote the recovery of upper-limb motor function in post-stroke patients. Increasing patients’ active participation by providing assist-as-needed (AAN) control strategies is key to the effectiveness of robot-assisted rehabilitation training. In this paper, a greedy assist-as-needed (GAAN) controller based on radial basis function (RBF) network combined with 3 degrees of freedom (3-DOF) potential constraints was proposed to provide AAN interactive forces of an end-effect upper limb rehabilitation robot. The proposed 3-DOF potential fields were adopted to constrain the tangential motions of three kinds of typical target trajectories (one-dimensional (1D) lines, two-dimensional (2D) curves and three-dimensional (3D) spirals) while the GAAN controller was designed to estimate the motor capability of a subject and provide appropriate robot-assisted forces. The co-simulation (Adams-Matlab/Simulink) experiments and behavioral experiments on 10 healthy volunteers were conducted to validate the utility of the GAAN controller. The experimental results demonstrated that the GAAN controller combined with 3-DOF potential field constraints enabled the subjects to actively participate in kinds of tracking tasks while keeping acceptable tracking accuracies. 3D spirals could be better in stimulating subjects’ active participation when compared to 1D and 2D target trajectories. The current GAAN controller has the potential to be applied to existing commercial upper limb rehabilitation robots.

## 1 Introduction

Stroke is one of the leading causes of long-term disability in adult persons worldwide (Veldema and Jansen, 2020). Accordingly, there is a growing demand for treatments of limb motor dysfunction in post-stroke patients. Nevertheless, due to the surge in the number of patients in the past decades, there is a rising demand for labor-intensive rehabilitation treatments, which has posed a significant burden on the national healthcare system (Johnson et al., 2019). To address this issue, robot-assisted training has become an important alternative in rehabilitation treatment. Rehabilitation robots can provide long-term and repetitive training sessions, customize different rehabilitation tasks according to the severity of a patient’s injury. Robot-assisted rehabilitation has become one of the important avenues to help post-patients restore their impaired limb functions and return to the community (Banala et al., 2010; Abdollahi et al., 2014; Ambrosini et al., 2017).

The control policies of rehabilitation robots can be divided into passive, assistive, and resistive modes (Kwakkel et al., 2008; Dalla Gasperina et al., 2021). Different from passive mode, the assistive and resistive modes require active participation of a patient in rehabilitation exercises. Previous literature has shown that for partially muscle-injured patients, the intrinsic drive of patients’ subjective movement intentions can generate plastic changes in the corresponding neural areas of the brain (Cauraugh and Summers, 2005; Stinear et al., 2008). Active patient participation is considered a crucial factor in fostering neural plasticity and facilitating movement recovery during rehabilitation treatment (Carr and Shepherd, 2010). To enhance patients’ engagement in rehabilitation training, an assist-as-needed (AAN) control strategy has been proposed. In the AAN policy, the controller not only tracks the target position but also adjusts the assisting torque based on a patient’s training performance (Taherifar et al., 2018; Zhong et al., 2020). With the AAN control scheme, the patients are able to independently perform prescribed tasks, and the rehabilitation robot can provide assistance only when deemed necessary. The AAN controller aims to offer minimal assistance and motivate a patient’s maximum active participation, accelerating the patient’s recovery process.

Prior studies have introduced AAN features into different rehabilitation robot control systems using different strategies (Asl et al., 2018; Asl et al., 2019; Lin et al., 2020). Emken et al. adopted a motion adaptation model to drive an adaptive robot controller, incorporating a spontaneously motivating forgetting factor to reduce assistance (Emken et al., 2007). Xiao et al. proposed an adaptive PID controller based on radial basis function (RBF) neural network (named RBF-PID) to improve the tracking performance (Xiao et al., 2023). The parameters of PID were updated by Jacobian matrix and RBF network using the movement errors between a patient’s healthy and the affected sides. For the estimation of a patient’s motor capability, the Gaussian RBF neural network has been extensively employed due to its property of universal approximation to any function (Girosi and Poggio, 1990). Since the motor capability of a patient with neurological injury may different in different workspace areas due to individual factors (e.g., fatigue, or spasms, etc.), the Gaussian RBF method was used with the assumption that the patient’s motor capability was directly related to the motion displacements of the rehabilitation robot (Sanner and Kosha, 1999).

It has been showed that an adaptive controller with Gaussian RBF was designed for the control of rehabilitation robot (Wolbrecht et al., 2008). To ensure a subject’s continuous active participation in the rehabilitation training, Wolbrecht et al. introduced a forgetting law to reduce the robot’s assistive force when the tracking error was below a certain threshold, effectively forgetting the previous estimation of motor capability. But this method had its limitations due to the interference from the forgetting factor. Ali Utku Pehlivan and colleagues proposed a minimal AAN control algorithm which could independently determine a subject’s motor capability at each moment using a Kalman filter (Pehlivan et al., 2016). However, the minimal AAN controller was constrained by the mechanical structure, and the computational complexity increased exponentially with the increase in mechanical degrees of freedom.

During rehabilitation training, the patients always tried to rely on the rehabilitation robot to provide as much assistive force as possible to complete the training task, inevitably leading to slackness and affecting the effectiveness of rehabilitation training (Reinkensmeyer et al., 2007). Nevertheless, based on the before-mentioned AAN control strategies (Li et al., 2022; Yang et al., 2023), when the rehabilitation robot detected a decrease of a patient’s exerted force, it was not able to determine whether the decrease was resulted from the objective lack of motor capability or subjective slackness. To solve this problem, Luo et al. proposed a greedy assist-as-needed (GAAN) algorithm, which could assess a subject’s motor capability by updating the weight vector of the RBF network (Luo et al., 2019). The GAAN control strategy enabled the RBF network to gradually learn the maximum force exerted by a patient over time. Recently, Pezeshki et al. (2023) presented an adaptive optimal control strategy to promote patients’ participation by modeling the interactive problem as a two-player non-zero-sum game.

Currently, the feasibility of GAAN control was verified on an upper limb end-traction rehabilitation robot with linear motion trajectories in a 2D plane. Tamantini et al. proposed a tunnel and back wall strategy to constrain the motions of different straight trajectories in 3D Cartesian space (Tamantini et al., 2023). Meanwhile, the existed researches adopted a unified control strategy to adjust a robot’s motion trajectories and enable the robot to interact with the physical environment (Khansari-Zadeh and Khatib, 2017; Feng et al., 2022). The unified control strategy could generate expected trajectory and control constraints using non-parametric potential functions and dissipative fields according to different rehabilitation tasks, and ensure the stability of robot-assisted rehabilitation training. Therefore, inspired by these studies on robot-assisted rehabilitation, it is essential to investigate that whether the GAAN control strategy can be effectively used for three-dimensional robot-assisted training tasks based on 3-DOF potential field constraints, since so far it has not been quantitatively evaluated on a rehabilitation robot.

In this paper, a 3-DOF potential field-constrained GAAN controller was proposed. The GAAN controller was designed by combining an AAN controller composed of RBF networks and a greedy algorithm to monitor the exerted forces of a subject and provide appropriate robot-assisted forces. The GAAN controller was tested with three types of typical training trajectories (one-dimensional lines, two-dimensional curves and three-dimensional spirals) via an end-effect upper limb rehabilitation robot. The proposed 3-DOF potential fields were adopted to constrain the tangential motions of three kinds of training trajectories. The effectiveness of the GAAN controller was verified both in the co-simulation (Adams-Matlab/Simulink) and subjects’ behavioral experiments. The outcomes indicate that the GAAN controller can be extended to kinds of three-dimensional robot-assisted rehabilitation.

## 2 Materials and methods

### 2.1 Upper-limb end-effect rehabilitation robot

**FIGURE 1:**
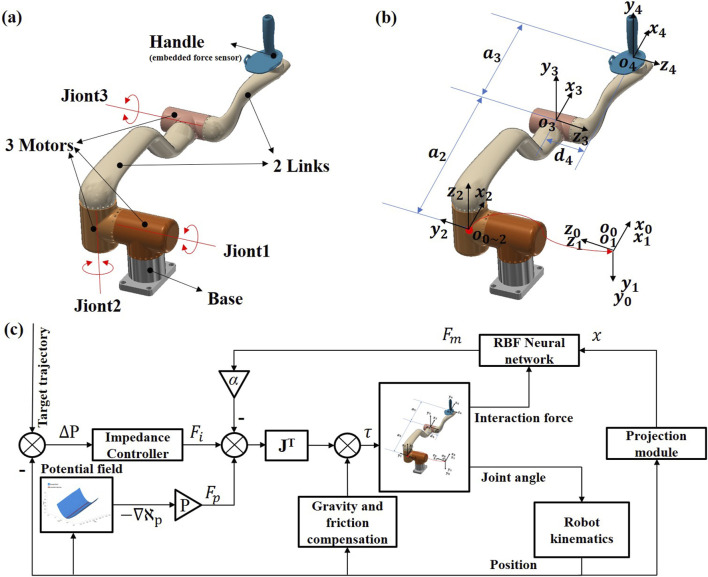
Diagram of 3-DOF upper limb end-effect rehabilitation robot (UERR) and the greedy assist-as-needed (GAAN) control framework. **(A)** Structure diagram. **(B)** Coordinate sketch-map and **(C)** The control framework of GAAN controller.

A 3-DOF upper-limb end-effect rehabilitation robot (UERR), consisted of two sets of symmetrical robotic arms, was adopted to evaluate the performance of the GAAN controller. The two robotic arms had symmetrical structure, and each arm was composed of a base, two links, three motors, and an end-effector handle. Motors were equipped with angle encoders for joint angle measurement, and a three-dimensional force sensor was embedded at the end-effector handle to collect force data during human-robot interactions. In current study, according to the handedness of a subject, only one robotic arm was adopted to perform the human-robot interactive task every time. The structure, coordinates and control framework of the GANN strategy are shown in Figures 1A–C, respectively.

### 2.2 Dynamic equations of UERR

The dynamic equation for the patient and robot system was given by:

$$M\left(q\right)\ddot{q}+C\left(q,\dot{q}\right)\dot{q}+G\left(q\right)q+f\left(\dot{q}\right)=\tau +{J\left(q\right)}^{T}F_{h}$$

where $q={\left[q_{1},q_{2},q_{3}\right]}^{T}$ is the matrix of three joint angles, $M\left(q\right)\in R^{3\times 3}$ denotes the inertia matrix, $C\left(q,\dot{q}\right)\in R^{3\times 3}$ represents the Coriolis and centrifugal matrix, $G\left(q\right)\in R^{3\times 3}$ is the gravity matrix, $f\left(\dot{q}\right)\in R^{3}$ is the friction torque matrix, and $\tau \in R^{3}$ represents the control torque matrix applied by the motors, $F_{h}\in R^{3}$ is the vector of forces applied by the patient on the end effector of the robot, and $J\left(q\right)\in R^{3\times 3}$ is the robot Jacobian matrix related with the joint angles. The Lagrangian method is employed in this paper to derive the matrices M, C, and G. The Stribeck friction torque model (Bo and Pavelescu, 1982) is utilized to identify the friction term $f\left(\dot{q}\right)$ . The definition of the robot additional coordinates is shown in Figure 1B.

### 2.3 Greedy assist-as-needed (GAAN) controller

#### 2.3.1 The control framework of GAAN

The key to AAN control lies in determining the optimal level of robotic assistance. This assistance should not be too minimal, in case it may result in significant tracking errors due to insufficient motor capability of some patients. Whereas, excessive assistance should be avoided to prevent a patient complacency and passive engagement in rehabilitation training (Pezeshki et al., 2023). Therefore, in order to provide appropriate robotic assistance and simultaneously activate a patient’s active participation, a GAAN controller was proposed, which mainly included two modules, i.e., an impedance controller module that was used to aid patients in completing movements and reducing tracking errors, and a Gaussian Radial Basis Function (RBF) network module that was used for modeling a patient’s motor capability and enabling GAAN control through a greedy-updating algorithm.

In the GAAN control, a patient user was required to perform a predefined human-robot interactive task by controlling the handle (i.e., end-effector) of the robot. The output force from the handle was mapped to the joint torque of the robot using the Jacobian matrix. The output force from the handle is represented as follow:

$$F_{r}=F_{i}-\alpha F_{m}$$

where $F_{r}$ represents the force provided by the robot in the end-effector space. $F_{i}$ and $F_{m}$ denote the assistive force from the impedance controller and the predicted force from a patient, respectively, as will be detailed in the following text. α is a scaling factor called challenge level that adjusts the task difficulty, weighting the forces exerted by the robot and the patient, and determining the level of assistance provided by the robot. Increasing the value of α will result in a decrease in the robot’s assistance level and an increase in the challenge level for the patient’s task, and *vice versa*.

Rehabilitation robot controllers typically employ a combination of impedance control and feedforward compensation (Ghannadi et al., 2018). The impedance controller is defined as follow:

$$F_{i}=K_{s}\left(x_{d}-x\right)-B_{d}\dot{x}$$ (1)

$${J\left(q\right)}^{T}F_{i}=\tau_{i}$$

where x and $x_{d}$ represent the actual position and desired position of the robot’s end-effector, and $\dot{x}$ denotes the velocity. $K_{s}$ and $B_{d}$ are the stiffness and damping of the robot’s impedance controller, respectively. $\tau_{i}\in R^{3}$ donates the torque produced by impedance controller in joint space.

In order to modeling a patient’s motor capability, the patient’s motor capability was generally assumed to directly related to his/her position in a task space (Wolbrecht et al., 2008). The Gaussian RBF network was often adopted to model a patient’s motor capability due to its good properties for approximating arbitrary functions (Girosi and Poggio, 1990). Different from previous studies that modeled a patient’s instantaneous power using RBF networks (Pehlivan et al., 2014), during rehabilitation training in the task space, the patient’s maximum exerted force will be progressively fitted using a Gaussian RBF network, as shown in the formula:

$$f_{m}=\Phi^{T}\omega$$

$$\Phi ={\left[g_{1}\;g_{2}\;g_{3}\ldots \ldots \;g_{n}\right]}^{T}$$

where $f_{m}$ is the magnitude of predicted force of a patient, *Φ* is an n × 1 vector containing radial basis functions. ω is a n-dimensional weight vector and can be updated by a greedy-updating algorithm (see 2.3.2). *n* is the nodes of RBFs along a motion trajectory, and to balance prediction accuracy and computational complexity, the number of RBFs is set to 20. $g_{i}$ represents the *i*th Gaussian RBF. The Gaussian RBF is defined as:

$$g_{i}=\exp\left(\frac{-{\left(x-\mu_{i}\right)}^{2}}{2\sigma^{2}}\right)$$

where $\mu_{i}$ is the center of the *i*th Gaussian RBF, *x* is the current value of a patient’s position, and σ is a scalar constant that determines the width of Gaussian RBFs. According to empirical experiments, the value of *σ* is set as $d/\sqrt{2n}$ , where d is the distance between the centers of the *1*st and *20*th radial basis functions.

#### 2.3.2 Greedy-updating algorithm based on RBF network

A patient’s maximum force over time was fitted through a Gaussian RBF network and then was used to update the GAAN controller. Calculation of the RBF network’s weight parameters can be divided into two parts, i.e., initialization and iteration. As the network update is related to the weight vector of the previous task, it is essential to obtain the initial weight vector of the RBF network. Hence, before the training begins, a patient was required to undergo training with only the baseline controller, so that the initial kinematic data and corresponding exerted force were recorded in real-time. Then, the recorded data was used to determine the initial weight vector for the RBF network based on least squares method.

$${H=\left[\begin{array}{ccc} \begin{array}{cc} g_{1,1} & g_{1,2}\\ g_{2,1} & g_{2,2} \end{array} & \cdots & \begin{array}{c} g_{1,m}\\ g_{2,m} \end{array}\\ \begin{array}{cc} \vdots & \vdots \end{array} & \ddots & \vdots \\ \begin{array}{cc} g_{n,1} & g_{n,2} \end{array} & \cdots & g_{n,m} \end{array}\right]}^{T}$$

$$F={\left[F_{s1}\;F_{s2}\;F_{s3}\ldots \;F_{sm}\right]}^{T}$$

$$\omega_{0}={\left(H^{T}H\right)}^{-1}H^{T}F$$

where *H* is an *n × m* matrix containing the time series of the Gaussian RBF network, where *m* and *n* denote the time series and the number of nodes, respectively. *F* is the time series of an *m × 1* vector, *F*_*sm*_ collected by the force sensor at the operation handle, where *m* represents the moment in time, and *f*_*m*_ is the data value of the force sensor at that moment. Since *f*_*m*_ *= Φ* ^*T*^ *ω*, the pseudo-inverse matrix ${\left(H\right)}^{T}H^{-1}H^{T}$ of matrix H is used to obtain *ω*. We obtain *ω*_*0*_ as the initial weight vector of the RBF network.

When ω_0_ is obtained, the weight vector ω is updated based on the following gradient descent algorithm

$$∆\omega =\frac{\lambda }{\sum_{0}^{t}L_{t}}\sum_{0}^{t}\max\left(0,F_{st}-F_{mt}\right)\Phi_{t}^{T}$$

$$L_{t}=\left\{\begin{array}{c} 1,\;if{\;F}_{st}>F_{mt}\\ 0,\;if{\;F}_{st}\leq F_{mt} \end{array}\right.$$

$$\omega_{k+1}=\omega_{k}+∆\omega$$

where λ is the learning rate, *t* is the sampling point in the task cycle, *k* is the task number, ∆ω is the average gradient over a task cycle, $\omega_{k}$ and $\omega_{k+1}$ are the weight vectors for the current and next tasks, respectively. *L*_*t*_ is a binary variable, the value is determined by comparing the measured value of the force sensor with the estimated value from the RBF network. If $F_{st}$ is greater than $F_{mt}$ , it indicates that the output of the RBF network is less than the maximum force exerted by the patient. Therefore, the weight vector needs to be updated to increase the output of the RBF network. Otherwise, when $F_{st}$ is less than $F_{mt}$ , it means that the patient should have the ability to exert greater force. Therefore, the control will maintain the weight vector to challenge the patient and encourage he/she to exert greater active force. Since the output of the RBF network is only updated in the increasing direction, the proposed robot-assisted strategy is called GAAN control. Moreover, if the patient occasionally reduces their exerted force due to relaxation or other subjective reasons, the GAAN controller will not misjudge a decrease in their functional ability, and it will not immediately increase robotic assistance.

#### 2.3.3 Projection module

It is widely known that the routine robot-assisted upper-limb rehabilitation tasks usually needs to be performed in 3D workspace. Thus, it is imperative to design a projection module to facilitate the RBF operation within a 3D workspace. The specific process is: first, the projection module obtains vector *V*_*1*_ by determining the start and end points of an interactive task; second, inputs the current position point and calculates the difference with the start point to obtain vector *V*_*2*_, and next derives the projected *x* as the input to RBF network using Equation 2. Meanwhile, we obtain the predicted force $F_{m}$ of a patient using Equation 3.

$$x=V_{1}\cdot V_{2}$$ (2)

$$F_{m}=V_{1}\cdot f_{m}$$ (3)

### 2.4 3-DOF potential fields in 3D workspace

The potential field was designed to prevent the motion trajectory deviating from the corresponding target trajectory. In current study, the potential field was applied to provide a predefined normal constraint force towards the target trajectory. The virtual potential field is designed as follows:

Uniformly sample *N* points from the designed expected trajectory:

$$D_{p}={\left\{p_{r}^{i}\right\}}_{i=1}^{N}$$

where $p_{r}^{i}\in R^{3}$ represents the position information of the end effector at the *i*th point in Cartesian space, i.e., a 3D column vector. $D_{p}$ denotes the discrete dataset of the expected trajectory at the end-effector.

Setting up the task space potential energy:

When a patient deviates from the expected trajectory, we expect the potential field to provide normal constraint forces, and the deviation error is linearly positively correlated with its normal constraint force. Inspired by Hooke’s law, we integrate the virtual spring force over distance to obtain the potential energy for each sampled point in the task space:

$$\varphi^{i}\left(p\right)=\varphi_{0}^{i}+\frac{1}{2}{K^{i}\left(p-p_{r}^{i}\right)}^{T}\left(p-p_{r}^{i}\right)\;\forall i\epsilon 1\ldots N$$ (4)

where, the potential energy $\varphi^{i}\left(p\right)$ for each point in the task space, with respect to the sampled points $p_{r}^{i}$ , is determined by *p* and $p_{r}^{i}$ . $\varphi_{0}^{i}$ is a scalar value, representing the initial potential energy at each sampled point. For a specific point *p*, there is a virtual spring between *p* and $p_{r}^{i}$ , and the attraction of $p_{r}^{i}$ to point *p* is given by $K^{i}\left(p-p_{r}^{i}\right)$ . Through integration, the elastic potential energy at point *p* is $\frac{1}{2}{K^{i}\left(p-p_{r}^{i}\right)}^{T}\left(p-p_{r}^{i}\right)$ . Therefore, the farther the point *p* from $p_{r}^{i}$ , the higher the elastic potential energy becomes, which aligns with the expected effect.

After obtaining $\varphi^{i}\left(p\right)$ , we introduce a weighting operator to control the influence of $p_{r}^{i}$ on point *p*. We use a Gaussian kernel function to calculate the potential energy weights from point *p* to the *N* sampled points:

$$w^{i}\left(p\right)=e^{-\frac{1}{{2\left(\sigma^{i}\right)}^{2}}{\left(p-p_{r}^{i}\right)}^{T}\left(p-p_{r}^{i}\right)}\;\forall i\epsilon 1\ldots N$$

where $\sigma^{i}$ determines the influence range of each sampled point and needs to be determined based on the specific rehabilitation task. Generally, we choose $\sigma^{i}$ so that at least 5% of the sampled points are within a distance of $1-\sigma^{i}$ from each center $p_{r}^{i}$ .

Normalize $w^{i}\left(p\right)$ , we obtain ${\tilde{w}}^{i}\left(p\right)$ :

$${\tilde{w}}^{i}\left(p\right)=\frac{w^{i}\left(p\right)}{\sum_{j}w^{j}\left(p\right)}\;\forall i\epsilon 1\ldots N$$

Multiplying ${\tilde{w}}^{i}\left(p\right){,\varphi }^{i}\left(p\right)$ then summing up, yields the total potential energy for each point in the task space:

$${ℵ}_{p}=\sum_{i}{\tilde{w}}^{i}\left(p\right)\varphi^{i}\left(p\right)$$

According to the principle of minimum energy, regardless of the state of the substance, it tends to transition towards the state with the lowest potential energy. Therefore, the negative gradient of the potential energy $-\nabla {ℵ}_{p}$ is calculated:

$$-\nabla {ℵ}_{p}=\sum_{i}\frac{1}{{\left(\sigma^{i}\right)}^{2}}{\tilde{w}}^{i}\left(p\right)\left(\varphi^{i}\left(p\right)-{ℵ}_{p}\right)\left(p-p^{i}\right)-{\tilde{w}}^{i}\left(p\right)K^{i}\left(p-p^{i}\right)$$ (5)

In specific rehabilitation tasks, the potential field mainly serves as a constraint in the normal direction. In the direction of motion, the robot-assisted force is calculated by the RBF network. Therefore, on the sampling dataset $D_{p}$ , we expect the gradient of the potential field $\gamma^{i}$ to be zero. To achieve this, it is necessary to optimize the potential field parameters $\varphi_{0}^{i}$ . This optimization can be formulated as solving a convex optimization problem, with the formula as follows:

$$minJ\left(\theta \right)=\frac{1}{N}\sum_{i=1}^{N}{\left\|\nabla {ℵ}_{p}\left(p_{r}^{i};\theta \right)+\gamma^{i}\right\|}^{2}$$

where, *θ* represents a vector composed of $\varphi_{0}^{i}$ , i.e., *θ =* $\left[\varphi_{0}^{1},\varphi_{0}^{2},\varphi_{0}^{3}\;\ldots \;\varphi_{0}^{N}\right]$ . Meanwhile, $\varphi_{0}^{i}\text{ and }\gamma^{i}$ are yield to the following conditional constraints:

$${0\leq \varphi }_{0}^{i}\;\forall i\epsilon 1\ldots N$$

$$\gamma^{i}=0\;\forall i\epsilon 1\ldots N$$

**FIGURE 2:**
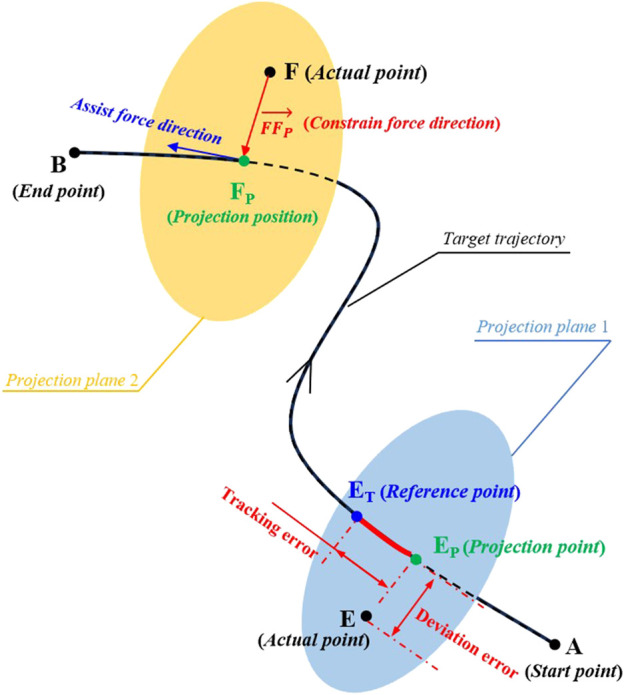
Diagram of a 3-DOF potential constraints and application of the robot-assisted force on a three-dimensional (3D) target trajectory. A and B are the starting and end points of the target tracking trajectory. E and F are the two different actual points of the end-effector of UERR at different tracking positions, and E_P_ and F_P_ are the respective projection points on the target trajectory, respectively. E_T_ is the reference point of the point E on the target trajectory. EE_P_ and E_T_E_P_ are the deviation error and tracking error of the tracking movements, separately.

In practice, the potential field constraints are designed according to specific target trajectories. In 3D workspace, the potential field is applied to provide normal constraint force perpendicular to the real-time motion direction a tracking movement, while the GAAN controller provides assisted force in the tangential direction of a tracking movement, see the details in Figure 2.

### 2.5 GAAN controller based on 3-DOF potential field constraints

In current study, the proposed GAAN controller based on 3-DOF potential field constraints aims to provide appropriate robotic assistance to the patients in 3D workspace. As shown in Figure 1C, the GAAN controller was constructed through a feed forward term, consisted of gravity compensation and friction compensation, as well as an output $F_{p}$ from the potential energy field, an output $F_{i}$ from the impedance controller and predicted force $F_{m}$ forming a feedback term. $F_{p}$ was obtained by multiplying the gradient of the potential energy field $-\nabla {ℵ}_{p}$ from Equation 5 by the mapping factor *P*. All the forces were computed in task space, and finally the forces were mapped to the joint space of the robotic arm through Jacobi matrix. The implement of potential field-based constraint and robot-assisted force is illustrated in Figure 2. For a specific interactive task, when the actual motion position (E) deviated the reference position (E_R_) on a target trajectory, the deviation distance between the actual motion position (E) and its projection point (Ep) on the target trajectory was defined as the deviation error, and the offset distance along the target trajectory between the projection point (Ep) and the reference position (E_R_) was regarded as the tracking error, respectively. Accordingly, the potential field constraint was proportionally applied along the deviation vector (*FF*_*p*_) in the form of a virtual spring force using Equation 4, and the robot-assisted force was exerted via GAAN controller along the tangential direction of the tracking movement, separately.

Three kinds of typical (linear, curvilinear, and spiral) potential field-constrained target trajectories were adopted to test the performance of the GAAN controller. The formula of the linear, curvilinear, and spiral trajectories are separately shown in Equations 6–8. Where x , y and z are the coordinates of the target trajectories in the base coordinate system, *X*_*ref*_ is a parameter between the range of 0–1, 0 and 1 correspond to the start point A and end point B of a target trajectory, respectively.

$$\left\{\begin{array}{c} x=0.753-0.861X_{ref}\\ y=-0.15-0.613X_{ref}\\ z=-0.06+0.325X_{ref} \end{array}\right.$$ (6)

$$\left\{\begin{array}{l} x=0.753-0.861X_{ref}\\ y=-0.15-0.613X_{ref}\\ \begin{array}{l} zt=-1+2X_{ref}\;\\ z=0.13827e^{zt}-0.1109 \end{array} \end{array}\right.$$ (7)

$$\left\{\begin{array}{l} t=2\pi X_{ref}\;\\ x=0.753-0.861X_{ref}+0.1\sin\left(t\right)\\ y=-0.15-0.613X_{ref}+0.1\sin\left(t\right)\\ zt=-1+2X_{ref}\;\\ z=0.13827e^{zt}-0.1109\; \end{array}\right.$$ (8)

### 2.6 Statistical analysis

All data were analyzed using IBM SPSS STATISTICS 23.0. Statistical tests (Kolmogorov-Smirnov) indicated that the data were not normally distributed and therefore non-parametric tests (cruskal-wallis rank sum tests) were employed to evaluate the statistically significant differences. Kruskal-Wallis H test was adopted to measure the significant influences of the training trajectory (linear, curvilinear, spiral) and challenge level ( α=0 , α=0.5 and α=1 ) on the tracking error blocks or the interactive force, respectively. A further pairwise comparison was measured with Nemenyi test (coding rank method) when requested. Moreover, for a certain factor (training trajectory or challenge level), Mann-Whitney U test was used to further examine the intra-group significance of the dependent variables if necessary. A p-value less than 0.05 was considered statistically significant.

## 3 Simulation validation

### 3.1 Co-simulation setup

A co-simulation (Adams-Matlab/Simulink) system was conducted to verify the theoretical feasibility of the GAAN controller based on 3-DOF potential field constraints. The control framework was established based on Matlab/Simulink with the numerical model exported from ADAMS software. Specifically, the virtual prototype (illustrated in Figures 1A, B) was imported to ADAMS, where the mechanical and physical environments, such as gravity, mechanical friction, impact forces and limitations were pre-configured according to requirements. With the control plugin in ADAMS, a numerical model, denoting the dynamics of the exoskeleton, could be exported as a Matlab/Simulink compatible module. With a sampling time of 1 ms, the stimulation model was able to accept the control signals generated by the control blocks and return the angular positions and velocities to Adams, displaying the simulation of the robot arm model in real-time.

In ADAMS, we set the material as aviation aluminum with the density of 2,730 kg/m^3^ and the gravity constant as 9.8 kg·m/s^2^, separately. The coulomb friction was applied to each joint where the static and dynamic friction coefficients were 0.3 and 0.12, respectively. The initial angles of each joint were [0°, 0°, 30°], and all the initial angular velocities were uniformly set to 0 rad/s. In order to simulate small scale disturbances in the stimulation environment, the disturbance signal was selected with a mean of 0 and a variance of 0.01 Gaussian signal, sampling at 20 Hz.

### 3.2 Simulation of potential field constraints

#### 3.2.1 Stimulation protocol

This simulation experiment was performed to simulate the constraint effects of the two levels of potential field constraints on the three types (linear, curvilinear, and spiral) of target trajectories. A reciprocating tracking task was conducted five times to verify the difference of the strong and weak potential field constraints. Two endpoints of the target trajectories were predefined according to the physical structure of UERR and pre-experimental tests of the subjects, where the joint angle coordinates corresponding to endpoints A and B were [0°, 0°, 30°] and [−86.8°, 5.2°, 28.9°] in the joint space of the UERR, respectively. The parameters of the strong and weak potential fields are K = 0.4 N/m, σ = 0.034, γ = 0, and K = 0.2 N/m, σ = 0.034, γ = 0, respectively. A PID controller (P = 300 N/m, D = 12 Ns/m, I = 0) was adopted to simulate of a patient’s force application on the end-effector of UERR. The reciprocal tracking speed of the controller for three types of target trajectories was uniformly set at 0.16 m/s. In addition, in order to simulate the irregular jitter of patients with neurological injuries during the tracking movement, a Gaussian disturbance force with a random direction and a mean value of 0 and a variance of 0.6 is applied to the end-effector of UERR and synchronously allocated to the respective joints of the robotic arm through inverse kinematics solution, with the updated rate of 1 Hz.

#### 3.2.2 Stimulation results

**FIGURE 3:**
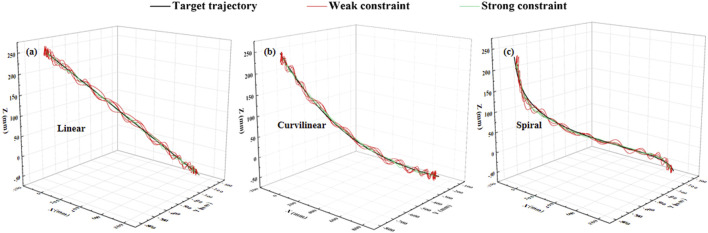
Stimulation demonstration of two levels of 3-DOF potential constraints (twice of forward and backward movements) on three kinds of typical [**(A)** linear, **(B)** curvilinear and **(C)** spiral] target trajectories (black color). Red and green curves indicate the weak and strong constraints, respectively.

**FIGURE 4:**
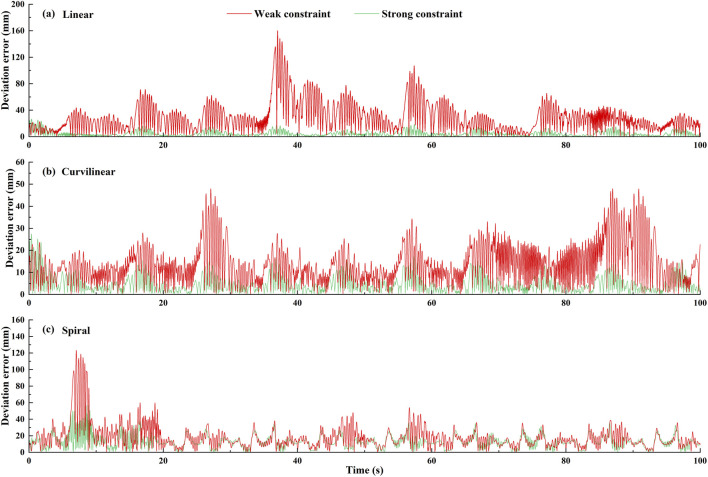
The dynamic deviation errors of two levels (weak/strong) of potential constraints on three types [**(A)** linear, **(B)** curvilinear and **(C)** spiral] of target trajectories over five times of stimulation reciprocating (forward and backward) movements.

The constraint effects of the two levels of potential fields on the three types (linear, curvilinear, and spiral) of target trajectories are shown in Figures 3, 4. For three types of target trajectories (black color), Figure 3 illustrated that the deviation errors with weak constraint (red color) were obviously greater than those with weak constraint (green color), respectively. Furthermore, the dynamic deviation errors of the three types target trajectories over the five times of reciprocating movements are displayed in Figure 4. It can be clearly shown that for linear and curvilinear trajectories, the average deviation errors (28.3 ± 20.7 mm and 13.9 ± 8.3 mm) with weak constraint were evidently bigger than those (4.5 ± 3.7 mm and 4.7 ± 3.6 mm) of the strong constraint (p < 0.001) (Figures 4A, B), while the difference (17.8 ± 14.2 mm and 12.9 ± 7.6 mm) with weak and strong constraints in spiral trajectory tend to reduce despite with significant difference (Figure 4C).

### 3.3 Simulation of impedance controller

#### 3.3.1 Stimulation protocol

This simulation experiment was performed to simulate the tracking ability of the proposed impedance controller. A reciprocating tracking task on three types (linear, curvilinear, and spiral) of target trajectories was carried on three times to verify the performance of the impedance controller. The impedance controller was defined by Equation 1, where its stiffness and damping were *Ks* = 260 N/m and *B*_*d*_ = 10 Ns/m. Variation of minimum jerk trajectory $X_{d}$ was adopted to quantify the tracking ability of the impedance controller

$$X_{d}=d*\left[10*{\left(\frac{t}{T}\right)}^{3}-15*{\left(\frac{t}{T}\right)}^{4}+6*{\left(\frac{t}{T}\right)}^{5}\right]$$

where, *X*_*d*_ is a minimum jerk trajectory of the three types of target trajectories. *T* represents the one-way cycle time of 5 s, a cycle time of a reciprocating movement is 10 s.*d* represents the actual tracking displacement of a target trajectory, which is uniformly set as 796 mm in current stimulation experiment.

#### 3.3.2 Stimulation results

**FIGURE 5:**
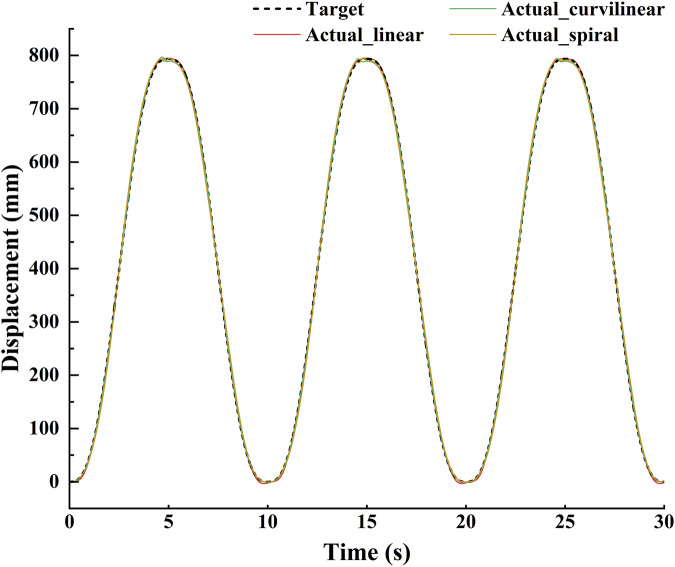
Demonstration of stimulation tracking effects of the impedance controller on the three types (linear, curvilinear and spiral) of target trajectories.

**FIGURE 6:**
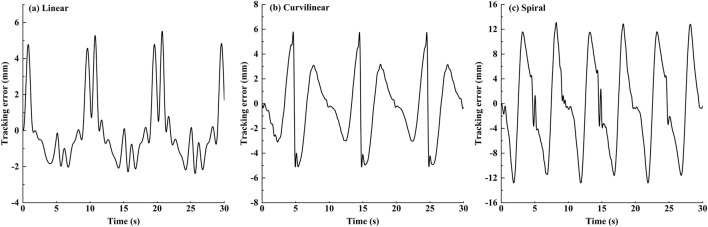
The dynamic stimulation tracking errors of the impedance controller on **(A)** linear, **(B)** curvilinear and **(C)** spiral trajectories, respectively.

The tracking effects of the impedance controller are shown in Figures 5, 6. The Figure 5 displayed that for three kinds of target trajectories, the controller maintained good dynamic tracking performances. In addition, the Figure 6 illustrated that the maximal tracking errors occurred mostly in the conversion time of the reciprocating movements, which were 5.9, 6.1 and 13.8 mm on linear, curvilinear and spiral trajectories, respectively. On the whole, the tracking error on spiral trajectory was significantly greater than those of the liner and curvilinear trajectories (p < 0.001).

### 3.4 Simulation of GAAN controller

#### 3.4.1 Stimulation protocol

This simulation is designed to verify the effectiveness of the proposed GAAN controller. We assumed that the initial output of the RBF neural network at each position point was 10N in the task space. Three levels of applied forces (i.e., 7N, 10N and 13N) added pseudorandom noise (mean ± variance = 0 ± 0.01) with an updated rate of 50 Hz were adopted to stimulate a patient’s force application during the robot-assisted rehabilitation exercises. The linear reciprocating tracking task with the tracking displacement of 796 mm (see details in 3.3) was performed ten times to test the performance of the GAAN controller. The output values of the RBF neural network relative to the respective applied forces were recorded to examine the control performance of the GAAN controller.

#### 3.4.2 Stimulation results

**FIGURE 7:**
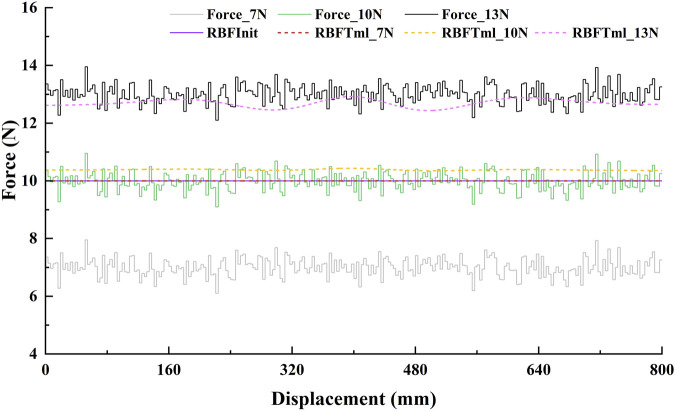
The greedy assist-as-needed stimulation effects of GAAN controller based on a linear trajectory (tracking displacement of 800 mm). Force_7N, Force_10N, and Force_13N are the three levels of stimulated (applied) force, and the RBFTm_7N, RBFTm_10N, and RBFTm_13N are the corresponding output forces of the GAAN controller, respectively. RBFInit is the initial output threshold of the GAAN controller.

The greedy AAN effects of the GAAN controller are shown in Figure 7. Based on the initial output force of 10N, it can be clearly shown that across the whole tracking displacement, the output values of the RBF network were 0 for applied force 7N, 10.3N for applied force 10N and fluctuations around 13N for applied force 13N, respectively. The overall output values of the RBF network indicate that the proposed GAAN controller was able to learn the applied forces, and only provide the appropriate force (difference between the applied force and the initial output force of the RBF network) when the applied force higher than the initial output force of the RBF.

## 4 Experiment validation

In order to further determine whether the GAAN controller can motivate a patient’s active participation and provide appropriate assistance in the robot-assisted rehabilitation training task. A battery of human evaluation tests were performed via the UERR-based experimental platform.

### 4.1 Experimental platform

**FIGURE 8:**
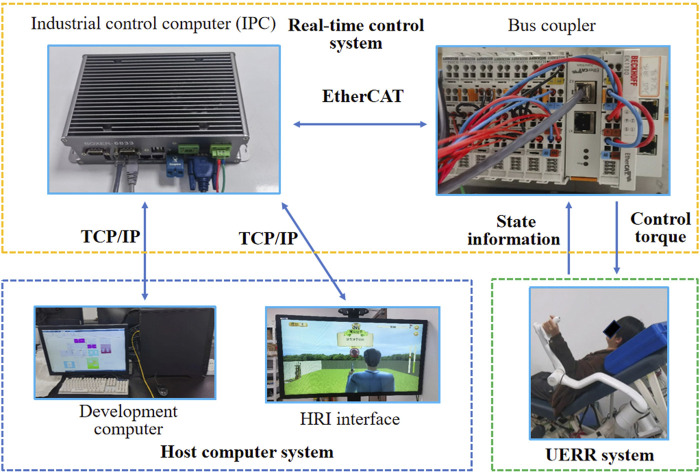
Display of UERR-based experimental platform.

The UERR-based experimental platform, consisted of a host computer system, real-time control system and UERR-based robotic arm, was adopted in current study. The host computer system was employed to run the Simulink programs and show an HRI interface used for guide the human-robot interactive tasks. The real-time control system was used to communicate between the host computer system and the UERR. Both the state information of the robotic arm and the master commends from the host computer could bi-directionally transmitted using industrial control computer (IPC) with a Bus coupler via TCP/IP and EtherCAT communication. The UERR-based robotic arm was adopted to execute kinds of robot-assisted interactive tasks with the sampling rate of 1000 Hz, as shown in Figure 8.

### 4.2 Subjects

10 healthy volunteers (6 males and 4 females, age 21–25 years) was recruited to participate in current study. All experiments are approved by the Ethics Committee of Ningbo Institute of Materials Technology and Engineering, Chinese Academy of Sciences. All subjects were shown the experimental procedure and provided informed consent before participation.

### 4.3 Experimental protocol

The objective of the human-robot interactive experiment was to verify that the current GAAN controller can promote subjects’ active participation and provide appropriate assistance in the robot-assisted rehabilitation exercises. In the experiment, each subject was asked to conduct a robot-assisted reciprocating tracking movement on three types (linear, curvilinear, and spiral) of target trajectories, under visual guidance of virtual reality (VR). Similar to the stimulation experiment, a reciprocating tracking movement included forward-and-backward displacements along the target trajectories of the robotic arm. Every subject was required to complete a reciprocating movement within 10 s, and a 3 s-break interval was given between the two forward and backward travels to avoid the fatigue effect. In order to verify the consistency of the results, all the control parameters were set the same as those in stimulation experiment (see details in *Simulation validation*). In view of different control abilities of the subjects in reciprocating tracking task, two RBF networks, independently trained with individual data, were used to model a subject’s motor capability in forward and backward movements, respectively.

Before the formal experiment, a preliminary experiment was performed to obtain the initial weight vectors of RBF networks, where the robotic device only adopted the baseline controller to provide the assistance, and the subject was required to apply his/her active force normally. The real-time position, velocity, and interactive force of the UERR were obtained and the initial weight vectors of RBF networks were determined with the least square method. During the formal experiment, each subject was asked to conduct three groups of reciprocating tracking task with three kinds of challenge level (α = 0, 0.5, 1) and the identical impedance controller. Each group of tracking task involved 10 sets of forward-and-backward movements. During the experiments, the subjects did not know the corresponding value of α, and the three groups was carried on in a random order. The whole experiment for each subject lasted about 1.5 h.

### 4.4 Experimental results

**FIGURE 9:**
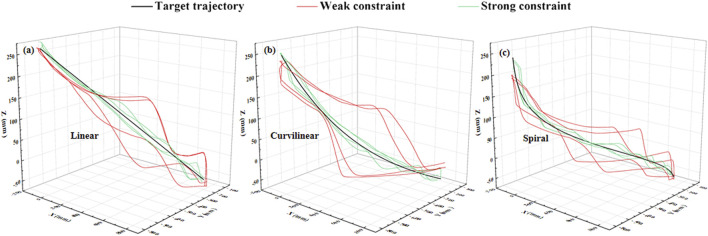
Experimental demonstration of two levels of 3-DOF potential constraints (twice of forward and backward movements) on **(A)** linear, **(B)** curvilinear and **(C)** spiral trajectories (black color). Red and green curves indicate the weak and strong constraints, respectively.

**FIGURE 10:**
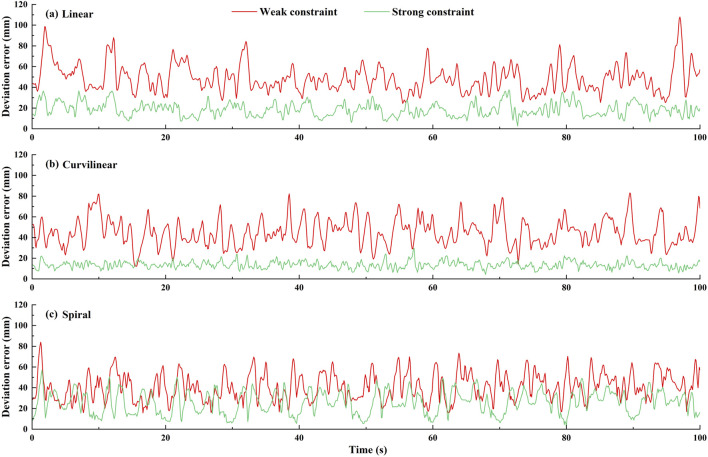
The dynamic deviation errors of **(A)** linear, **(B)** curvilinear and **(C)** spiral trajectories under two levels (weak and strong) of potential constraints across 10 subjects.

Figures 9A–C shows an example of the constraint effects with two levels of potential fields on the three types (linear, curvilinear, and spiral) of target trajectories. It can be clearly showed that with two sets of forward and backward movements, the deviation errors with weak constraint were obviously greater than those with weak constraint, respectively. Furthermore, the dynamic deviation errors of the three types target trajectories over the 10 times of reciprocating movements are displayed in Figure 10. It can be clearly shown that for linear and curvilinear trajectories, the average deviation errors (48.8 ± 13.3 mm and 445.1 ± 12.7 mm) with weak constraint were evidently bigger than those (18.6 ± 6.4 mm and 13.5 ± 3.4 mm) of the strong constraint (p < 0.01) (Figures 10A, B), while the difference (40.9 ± 12.9 mm and 25.9 ± 10.8 mm) with weak and strong constraints in spiral trajectory tend to reduce despite with significant difference (Figure 10C).

**FIGURE 11:**
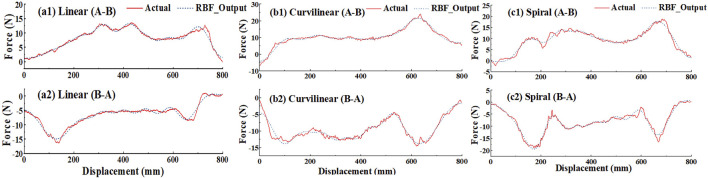
Approximation of RBF networks and the actual measurement about the interactive forces on the end effector of the robot based on linear, curvilinear and spiral target trajectories, respectively. ‘Actual’ and ‘RBF_output’ represent the actual force applied by a subject and the corresponding RBF networks output, respectively. Figures (a1), (b1), and (c1) denote the measurements are in forward (A–B) movements, and Figures (a2), (b2), and (c2) are denote the measurements are in backward (B–A) movements, respectively.

Figure 11 shows the RBF estimated interactive forces and the actual interactive forces on three types (linear, curvilinear and spiral) of target trajectories. It was observed that the trends of the force across the tracking displacement were significantly different between the forward (A-B) and backward (B-A) movements. For the forward (A-B) and backward (B-A) movements, the RMSEs between RBF estimated force and actual force were 0.35 (Figure 11a1) and 0.34N (Figure 11a2) on linear trajectory, 0.36 N (Figure 11b1) and 0.36 N (Figure 11b2) on curvilinear trajectory and 0.41 N (Figure 11c1) and 0.38 N (Figure 11c2) on spiral trajectory, respectively.

**FIGURE 12:**
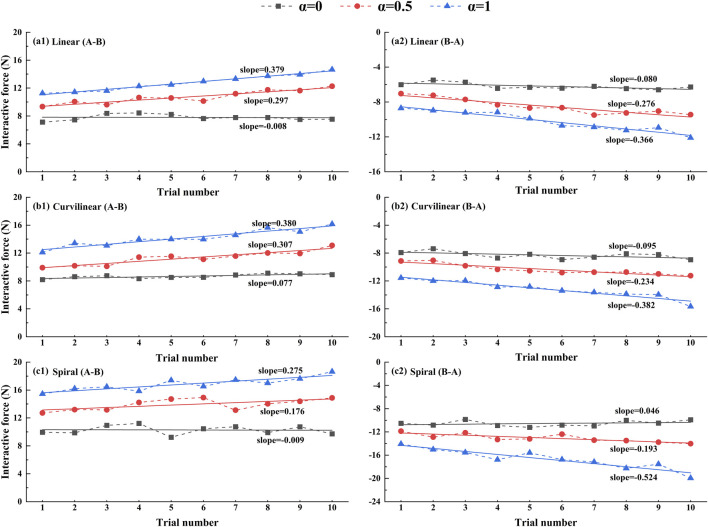
Trends of average interactive forces of subjects (N = 10) against the trial numbers of forward (A–B) and backward (B–A) movements. A first-order linear function is used to fitting the relation between the average force and trial number. Figures (a1), (b1), (c1) present the results of linear, curvilinear and spiral trajectories in forward (A–B) movements, respectively. Figures (a2), (b2), (c2) present the results of linear, curvilinear and spiral trajectories in backward (B–A) movements, respectively. α = 0, α = 0.5 and α = 1 represent three kinds of task challenge levels.

Figure 12 shows the trends of the interactive force on three types (linear, curvilinear and spiral) of target trajectories with three levels of challenge levels (α = 0, 0.5, 1) over the sequential tracking (forward (A-B) and backward (B-A)) movements. With the increase of the tracking trials, the interactive forces based on three levels of challenge levels displayed consistent upward trends in the forward (A-B) movements and downward trends in the backward (B-A) movements, separately. For challenge levels α = 0, α = 0.5 and α = 1, the upward slopes of the force trend were 0.379, 0.297, and 0.008 on linear trajectory, 0.380, 0.307, and 0.077 on curvilinear trajectory, and 0.275, 0.176, and 0.009 on spiral trajectory, in the forward (A-B) movements, respectively. Accordingly, the downward slopes of the force trend were −0.080, −0.276 and −0.366 on linear trajectory, −0.095, −0.234 and −0.382 on curvilinear trajectory, and −0.046, −0.193 and −0.524 on spiral trajectory, in the backward (B-A) movements, respectively.

**FIGURE 13:**
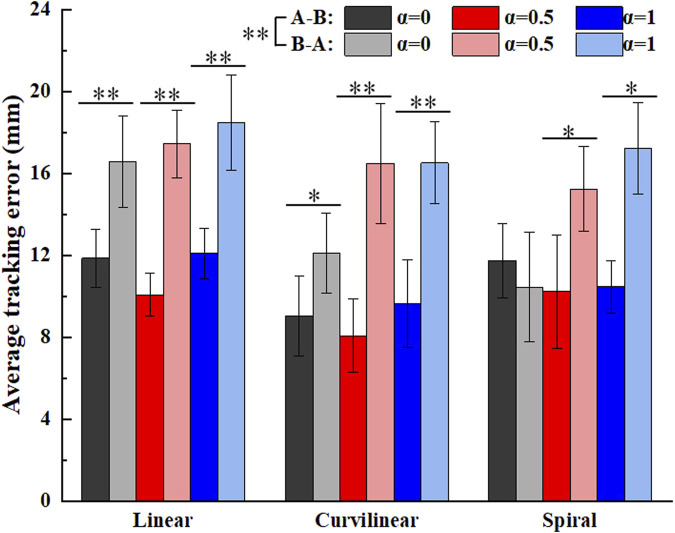
Average tracking errors of subjects (N = 10) on three kinds of target trajectories with three levels of task challenge levels (α = 0, α = 0.5 and α = 1). ‘A-B’ and ‘B-A’ signify the forward and backward movements, respectively. ‘*’ and ‘**’ represent the significant difference with the significance level less than 0.05 and 0.01, respectively.

Figure 13 shows the average tracking errors on three types (linear, curvilinear and spiral) of target trajectories with three kinds of challenge levels (α = 0, 0.5, 1) in forward (A-B) and backward (B-A) movements. The Kruskal-Wallis H test illustrated that there was significant difference between the forward (A-B) and backward (B-A) movements (p < 0.01), but not differences displayed among the three types of tracking trajectories. For challenge levels α = 0, α = 0.5 and α = 1, two kinds of tracking errors in A-B and B-A movements were 11.9 ± 1.4 mm and 16.6 ± 2.2 mm, 10.1 ± 1.0 mm and 17.4 ± 1.7 mm, and 12.1 ± 1.2 mm and 18.5 ± 2.3 mm on linear trajectory, 9.0 ± 1.9 mm and 12.1 ± 1.9 mm, 8.1 ± 1.8 mm and 16.5 ± 2.9 mm, and 9.6 ± 2.1 mm and 16.5 ± 2.0 mm on curvilinear trajectory, 11.7 ± 1.8 mm and 10.4 ± 2.7 mm, 10.2 ± 2.8 mm and 15.2 ± 2.1 mm, and 10.5 ± 1.3 mm and 17.2 ± 2.2 mm on spiral trajectory, respectively. The Mann-Whitney U tests further indicated that there were significant differences of the two kinds of tracking errors in A-B and B-A movements (p < 0.05) with exceptions of the two paired groups (curvilinear and α = 0, spiral and α = 1).

**FIGURE 14:**
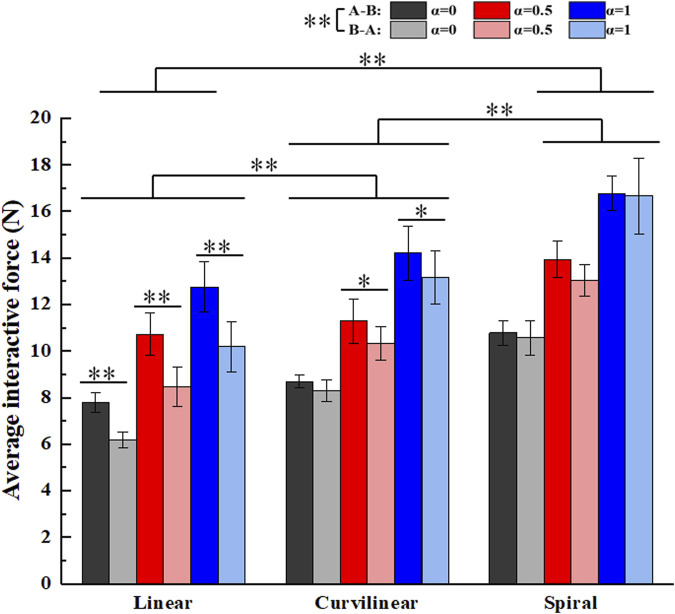
Average interactive forces of subjects (N = 10) on three kinds of target trajectories with three levels of task challenge levels (α = 0, α = 0.5 and α = 1). ‘A-B’ and ‘B-A’ signify the forward and backward movements, respectively. ‘*’ and ‘**’ represent the significant difference with the significance level less than 0.05 and 0.01, respectively.

Figure 14 shows the average interactive forces on three types (linear, curvilinear and spiral) of target trajectories with three kinds of challenge levels (α = 0, 0.5, 1) in forward (A-B) and backward (B-A) movements. The Kruskal-Wallis H test displayed that there were significant differences between the forward (A-B) and backward (B-A) movements (p < 0.01), and among the three types of target trajectories (p < 0.01), respectively. For challenge levels α = 0, α = 0.5 and α = 1, two kinds of interactive forces in A-B and B-A movements were 7.8 ± 0.4N and 6.2 ± 0.3N, 10.7 ± 0.9N and 8.5 ± 0.8N, and 12.8 ± 1.1N and 10.2 ± 1.1N on linear trajectory, 8.7 ± 0.3N and 8.3 ± 0.5N, 11.3 ± 0.9N and 10.3 ± 0.7N, and 14.2 ± 1.2N and 13.2 ± 1.1N on curvilinear trajectory, 10.8 ± 0.5N and 10.6 ± 0.7N, 13.9 ± 0.8N and 13.0 ± 0.7N, and 16.8 ± 0.7N and 16.7 ± 1.6N on spiral trajectory, respectively. The Mann-Whitney U tests further showed that there were significant differences of the two kinds of interactive forces in A-B and B-A movements on linear trajectory (p < 0.01) and two paired groups (α = 0 and α = 0.5) on curvilinear trajectory (p < 0.05), respectively.

**FIGURE 15:**
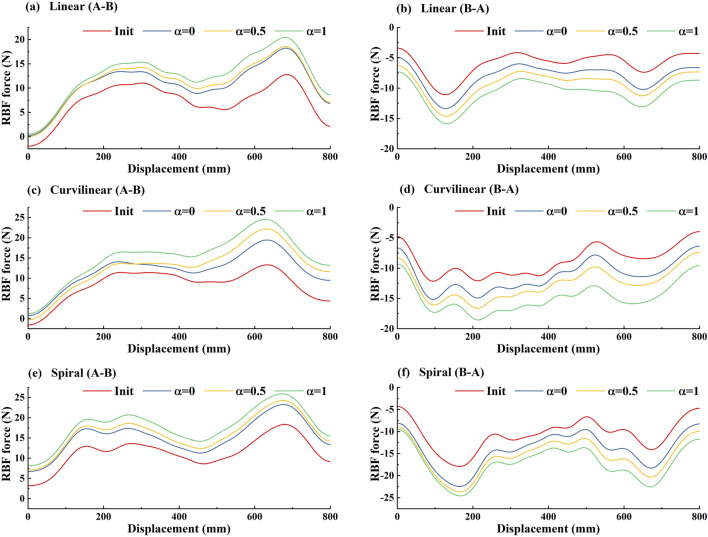
The average outputs of forward (A-B) and backward (B-A) RBF networks across 10 subjects on three kinds [**(A, B)** linear, **(C, D)** curvilinear and **(E, F)** spiral] of target trajectories with three levels of task challenge levels. ‘A-B’ and ‘B-A’ signify the forward and backward movements, respectively. 'Init' represents the initial RBF networks and ‘α = 0’, ‘α = 0.50’ and ‘α = 1’ represent three levels of task challenge levels, respectively.

In order to further confirm that the subjects’ active participation can be encouraged by the GAAN controller, the average outputs of the forward (A-B) and backward (B-A) RBF networks across the subjects are presented in Figure 15. The results showed that based on linear, curvilinear and spiral trajectories, the overall trends of the RBF force in forward (A-B) movements (Figures 15A, C, E) were evidently different those in backward (B-A) movements (Figures 15B, D, F). As the challenge level orderly increased from α = 0, 0.5 to 1, the forward (A-B) and backward (B-A) RBF forces were larger than the respective initial RBF outputs and increased over the tracking displacements accordingly. Since the weight vectors of RBF networks only updated when the motor capability of subjects was more than the outputs of RBF networks, the increasing trends of RBF forces reflected the improvement of the subjects’ active effort. Therefore, the subjects’ active participation was indeed stimulated with the task challenge provided by RBF networks.

## 5 Discussion

This study presents a novel GAAN controller based on 3-DOF potential constraints for the upper limb rehabilitation training of the patients with limb dysfunction. The GAAN controller is designed to estimate the motor capability of a subject and provide appropriate robot-assisted forces by combining an impedance controller and RBF networks. The appropriate assistance for participants was implemented from two aspects. The robot-assisted forces corresponding to workspace position are regulated based on the functional capability approximated by RBF networks. Synchronously, the assisted forces are influenced by the target task and the task challenge level, which is modified according to the task performance of subjects. Distinguished from the general kind of AAN controller (e.g., an improved PID controller (Xiao et al., 2023)), the greedy strategy is implemented by timely updating a subject’ motor capability model, which stimulates the subject to keep continuous active participation. Thus, the GAAN controller can promote the subjects’ active engagement during training exercises, and thus improving the outcome of rehabilitation therapy (Luo et al., 2019).

To verify the utility of the proposed GAAN controller on UERR, three types (linear, curvilinear and spiral) of target trajectories with two levels of 3-DOF potential constraints and three kinds of task challenge levels were adopted to validate the performance of the proposed GAAN controller. The co-simulation (Adams-Matlab/Simulink) experiments indicated that the GAAN controller maintained good tracking ability (Figures 5, 6) and GAAN ability (Figure 7). Furthermore, the results of Figure 11 showed that the RBF estimated interactive forces could accurately estimate the actual measured interactive forces. The influence of different task challenge levels (α = 0, 0.5 and 1) on the interactive forces and the RBF forces were investigated in the forward and backward movements. It can be showed that as the number of training trials increases, the average interactive forces of subjects gradually increases (Figure 12), and the higher challenge levels correspond to higher interactive forces and RBF forces (Figures 12, 15). It indicates that a relatively higher challenge level provided by the GAAN controller can enhance the subjects’ active participation during the training process.

Moreover, the tracking errors of a target trajectory can provide important reference to the effect of a patient’ rehabilitation training. Luo et al. and Tamantini et al. both proposed robot-assisted control strategies, the target trajectories adopted in their studies are one-dimensional straight movements in task space (Luo et al., 2019; Tamantini et al., 2023). Pezeshki et al. presented an adaptive optimal control strategy to promote patients’ participation (Pezeshki et al., 2023), the target trajectory in the study was a two-dimensional circular curve. Therefore, the average tracking errors and the average interactive forces based on three types (linear, curvilinear and spiral) of target trajectories with three kinds of challenge levels were calculated to verify the performance of the GAAN controller. The results showed that there were no significant differences of the average tracking errors among the linear, curvilinear and spiral trajectories (Figure 13). It is probably that the tracking tasks with current target trajectories are not challenge enough for healthy subjects. It is also worthy of further study in post-stroke patients. In addition, Figure 14 illustrated that there were significant differences of the average interactive forces among the linear, curvilinear and spiral trajectories, respectively. It can be interpreted that the relatively complex target trajectories (e.g., curvilinear and spiral) required the subjects put much more efforts to fulfill the tracking movements, while maintaining undifferentiated tracking errors.

From the above results, it can be concluded that the proposed GAAN controller has the potential to be directly applied to the commercial rehabilitation robots. Although the experimental results demonstrate that the 3D spiral trajectory is superior to the 1D or 2D trajectory, it still needs to combine more 3D target trajectories with VR training scenarios to achieve the optimal rehabilitation training effect. In the future, the above conclusions should be verified from two aspects: 1) integrating the GAAN algorithm into other 7-DOF bilateral rehabilitation robots; 2) Recruit more post-stroke patients to further validate its effectiveness.

## 6 Conclusion

A GAAN controller based on 3-DOF potential field constraints was proposed to provide AAN interactive forces via a 3-DOF EURR. Co-simulation experiments and behavioral experiments on 10 healthy volunteers are carried out to verify the effectiveness of the GAAN. It was verified by experiments that: 1) the proposed GAAN controller indeed enhances the subjects’ active participation during the training process; 2) Compared to 1D linear and 2D curvilinear trajectories, 3D spiral trajectory enables the subjects devote much more efforts to tracking task, stimulating subjects to keep continuous active participation. The focus of future work will be introducing psychological and physiological measurements to further determine the subjects’ active participation in rehabilitation training.

## Data Availability

The raw data supporting the conclusions of this article will be made available by the authors, without undue reservation.
